# Novel approach to optimization of Alzheimer’s vaccine configuration for maximal targeting of toxic amyloid‐beta oligomers

**DOI:** 10.1002/alz.086601

**Published:** 2025-01-09

**Authors:** Johanne Kaplan, Ebrima Gibbs, Scott Napper, Erin Scruten, Juliane Coutts, Neil R Cashman

**Affiliations:** ^1^ ProMIS Neurosciences, Toronto, ON Canada; ^2^ University of British Columbia, Vancouver, BC Canada; ^3^ University of Saskatchewan, VIDO, Saskatoon, SK Canada

## Abstract

**Background:**

A large body of evidence now indicates that the most pathogenic species of Aß in Alzheimer’s disease (AD) consist of soluble toxic oligomers (AßO) as opposed to insoluble fibrils and monomers. Using our computational platform, we identified 4 different AßO‐restricted conformational B cell epitopes (300, 301, 303, 305) that were tested as vaccines for their ability to induce an antibody response that selectively targets toxic AßO, without inducing potentially detrimental B or T cell responses against plaque or normal Aß. A novel ex vivo approach was then used to select an optimal vaccine configuration amongst the 15 possible combinations of the 4 epitopes to provide maximal binding to a toxic oligomer‐enriched low molecular weight (LMW) fraction of soluble AD brain extracts.

**Method:**

Mice were vaccinated with four different AßO‐restricted conformational B cell epitopes conjugated to KLH to provide T cell help and formulated with QS‐21 adjuvant. Serum IgG titers against the peptide epitopes were measured by ELISA and T helper cell responses by ELISPOT. The reactivity of serum antibodies with AßO versus monomers was evaluated by SPR, and plaque binding by immunohistochemistry.

**Result:**

All 4 epitopes elicited robust antibody responses when administered either individually or together as part of a quadrivalent vaccine. ELISPOT analysis showed a T cell response to KLH and not the AßO B cell epitopes. Serum antibodies elicited by the monovalent and quadrivalent vaccines all showed the desired selectivity and reacted with AßO only, not monomers nor plaque. Comparison of the SPR binding responses to the toxic AßO‐enriched LMW fraction of AD brain extract by equivalent amounts of IgG from immune serum of monovalent vaccines vs mixtures of 2, 3 or 4 sera was used to rank vaccine configurations. Interestingly, maximal reactivity was observed with immune IgG against the monovalent vaccine containing epitope 301, the target of PMN310, our clinical‐stage monoclonal antibody. There was no advantage of additional epitopes beyond 301 alone.

**Conclusion:**

Vaccination with AßO‐restricted conformational B cell epitopes conjugated to KLH produced strong antibody responses with no measurable pro‐inflammatory T cell responses against Aß. Immunization with epitope 301 alone was sufficient to produce maximal reactivity against brain AßO.

